# Accelerometer-Derived Rest-Activity Rhythm Amplitude, Genetic Predisposition, and the Risk of Ischemic Heart Disease: Observational and Mendelian Randomization Study

**DOI:** 10.2196/79301

**Published:** 2025-12-15

**Authors:** Lele Wang, Juying Zhang, Xiong Xiao, Xuewei Tang, Jianxiang Tang, Hao Xu, Bing Guo, Xing Zhao

**Affiliations:** 1 West China School of Public Health and West China Fourth Hospital Sichuan University Chengdu China; 2 West China Hospital of Stomatology State Key Laboratory of Oral Diseases, National Clinical Research Center for Oral Diseases, Research Unit of Oral Carcinogenesis and Management, Chinese Academy of Medical Sciences Sichuan University Chengdu, Sichuan China

**Keywords:** rest-activity rhythm amplitude, ischemic heart disease, genetic predisposition, Mendelian randomization, physical activity

## Abstract

**Background:**

The rest-activity rhythm amplitude (RARA), as a fundamental human behavior, has been linked to various health conditions. However, its causal relationship with ischemic heart disease (IHD), along with the potential modification by genetic predisposition, remains unclear.

**Objective:**

This study aimed to investigate the causal association between RARA and IHD using a triangulation approach that incorporated both observational and Mendelian randomization (MR) analyses, and to determine whether genetic predisposition modifies this relationship.

**Methods:**

First, a prospective cohort analysis was conducted among individuals who had no history of IHD before wearing wrist actigraphy between 2013 and 2015 in the UK Biobank. RARA was derived nonparametrically from accelerometer data worn for at least 7 days. Disrupted RARA was established as the lowest quintile of accelerometer-derived amplitude. Incident IHD was identified through medical records using *ICD-10* (*International Statistical Classification of Diseases, Tenth Revision*) codes I20-25. Genetic predisposition was assessed with polygenic risk scores for IHD (IHD-PRS), which were categorized into “low IHD-PRS” (lowest quartile), “intermediate IHD-PRS” (second and third quartiles), and “high IHD-PRS” (highest quartile). Cox proportional hazards models were used to assess the association between RARA and incident IHD, as well as the modification effects of IHD-PRS. Second, we obtained RARA genome-wide association study data from the UK Biobank and IHD genome-wide association study data from FinnGen. A 2-sample MR using inverse-variance weighted methods was performed to examine the causality between them. Several other well-established methods, including random-effects and radial inverse-variance weighted method, Mendelian randomization pleiotropy residual sum and outlier, and maximum likelihood, were also performed for sensitivity analyses.

**Results:**

A total of 84,095 participants were followed up for a median of 7.90 (IQR 7.33-8.41) years. Overall, 3870 (4.60%) individuals developed IHD. Disrupted RARA was significantly associated with a higher risk of IHD (hazard ratio [HR] 1.20, 95% CI 1.12-1.30; *P*=.002). No significant modification effects by genetic predisposition on the multiplicative scale were found for this association (HR 0.92, 95% CI 0.76-1.11; *P*=.39 and HR 0.91, 95% CI 0.74-1.12; *P*=.37, respectively). The results remained consistent when we used the additive interaction scale to assess effect modification. Compared with participants with high RARA and low IHD-PRS (reference), those with disrupted RARA and high IHD-PRS had the highest risk of IHD (HR 2.63, 95% CI 2.29-3.02; *P*<.001), while those with disrupted RARA and low IHD-PRS had the smallest increased risk (HR 1.29, 95% CI 1.10-1.52; *P*<.001). The remaining groups showed intermediate risks in ascending order. MR results supported the observational findings (odds ratio [OR] 1.13, 95% CI 1.00-1.28; *P*=.047). This association was robust in our sensitivity MR analyses.

**Conclusions:**

The study suggests a potential causal relationship between RARA and IHD, independent of genetic predisposition, highlighting the significance of RARA for IHD prevention.

## Introduction

Ischemic heart disease (IHD), as one of the most prevalent noncommunicable chronic diseases, is now the number 1 cause of global mortality and a major contributor to disability [1-3]. The latest research shows that IHD has the highest global age-standardized disability-adjusted life years of all diseases at 2275.9 per 100,000 and causes 108.8 deaths per 100,000 [1]. Despite the awareness that IHD can be prevented by appropriate lifestyle and behavioral interventions, the global progress against IHD is flatlining and even shows signs of reversing in some areas [2,4,5]. Identifying new modifiable risk factors for IHD is therefore imperative.

The rest-activity rhythm amplitude (RARA), encompassing the dynamic physiological and behavioral variations in physical activity, sleep duration, and sedentary behaviors over 24 hours, significantly contributes to the overall equilibrium of one’s physical and mental well-being [6,7] (eg, type 2 diabetes [8,9], atrial fibrillation [10], and stroke [11]). As a biomarker that can be objectively measured and easily intervened upon, previous studies have initially explored the relationship between RARA and IHD. Although they were limited by small sample sizes [12-14], a cross-sectional study [13] and a limited set of populations [12,14], these studies provide some mechanistic pathways that may apply to the general population, such as affecting energy and hormones, leading to metabolic abnormalities and inflammatory responses, which can lead to adverse cardiovascular consequences. Furthermore, it is well known that genetic predisposition plays a significant role in incident IHD [15]. The investigation of gene-behavior interactions can provide novel insights into the precise prediction of and targeted interventions for cardiovascular disease. However, there is no study that has explored the potential modifying effects of genetic predisposition on such a relationship.

In addition, it is difficult to assess the causal relevance using observational evidence alone due to reverse causality and confounding [16-18]. Moreover, because of ethical issues, it is impossible to design randomized clinical trials in the population to intervene in a proportion of individuals to disrupt their rhythms and thus explore the causal effect. Mendelian randomization (MR) identifies causal effects by using a genetic instrument that relies on randomly and independently distributed genetic variants during meiosis while avoiding ethical concerns. This methodology has gained widespread recognition for uncovering pivotal causal factors associated with cardiovascular diseases [19,20].

Therefore, we used a triangulation approach that integrates different epidemiological methods to (1) assess the associations between RARA and incident IHD among nearly 85,000 participants of the UK Biobank prospective cohort study, and determine whether genetic predisposition modifies such association, and (2) examine the causality between RARA and IHD using 2-sample MR. Through these comprehensive analyses, our overall goal is to identify whether RARA is a new intervenable risk factor for IHD.

## Methods

### Study Design

**Figure 1:**
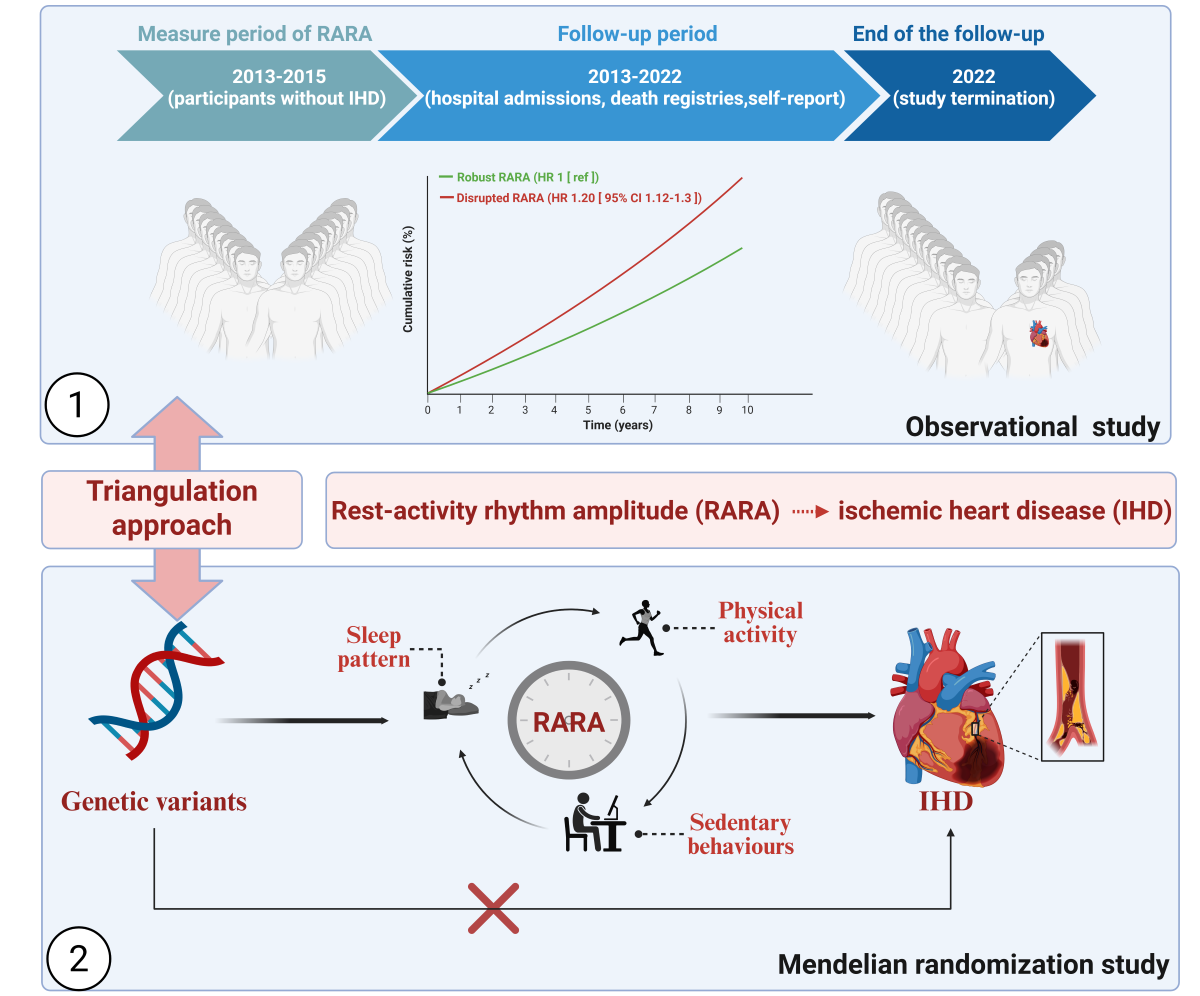
Study design for the triangulation approach of rest-activity rhythm amplitude (RARA), genetic predisposition, and ischemic heart disease (IHD).

Figure 1 outlines the overall study design. In summary, the study used a triangulation framework that integrated both observational and MR analyses to address a central question: whether there is a causal relationship between RARA and IHD.

### Prospective Cohort Study

The UK Biobank, an ongoing and extensive population-based prospective cohort study, enrolled 502,629 participants aged 40-69 years living in England, Scotland, or Wales between 2006 and 2010. It collected multidimensional information from participants encompassing environmental exposures, genetics, bio samples, and health outcomes, and continued longitudinal follow-up. Detailed information about the UK Biobank can be found online. Data from a substudy (over 100,000 individuals wearing an Axivity AX3 wrist-based triaxial accelerometer continuously for 7 days from 2013 to 2015) were used in this study.

The exclusion criteria were defined as follows: (1) participants identified by the UK Biobank accelerometer working group as having unreliable accelerometer data (indicated by field ID 90002, 90015, 90016, 90017, 90180, and 90182). Refer to the methods section of Multimedia Appendix 1 for more detailed data quality metrics, (2) individuals who were diagnosed with IHD based on self-report history or medical records with *ICD-10* (*International Statistical Classification of Diseases, Tenth Revision*) codes before wearing the accelerometer, and (3) participants with missing covariates. Finally, 84,095 participants were included in our analysis. The detailed flowchart for participant inclusion is provided in Figure S1 in Multimedia Appendix 1.

### Mendelian Randomization Study

The datasets used in this research were sourced from publicly accessible repositories, and all original papers obtained ethical approvals. Single-nucleotide polymorphisms (SNPs) related to RARA were provided by a genome-wide association study (GWAS) of 27 accelerometry-derived physical activity and RARA measurements from the UK Biobank (n=88,411) [21]. We obtained the summary statistics for IHD from FinnGen [22] of 500,348 individuals. More details are provided in Table S1 in Multimedia Appendix 1 and the original paper.

### Rest-Activity Rhythm Amplitude Measurement

A total of 106,053 individuals in the UK Biobank agreed and were provided with a wrist-worn Axivity AX3 accelerometer from 2013 to 2015. This device, set to activate at 10 AM, 2 working days after being dispatched, recorded triaxial acceleration data for 7 days at 100 Hz, with a dynamic range of ± 8 gravity (9.8 m/s^2^). Participants were instructed to wear the accelerometer on their dominant wrist continuously throughout their usual activities. For the effective usage of the nonwear data, the UK Biobank accelerometer working group imputed nonwear time data using linear interpolation [23], and the average imputed ratio is 4.28% in our analysis. More details about accelerometer data processing, flagging, and summarizing have been published [23].

**Figure 2:**
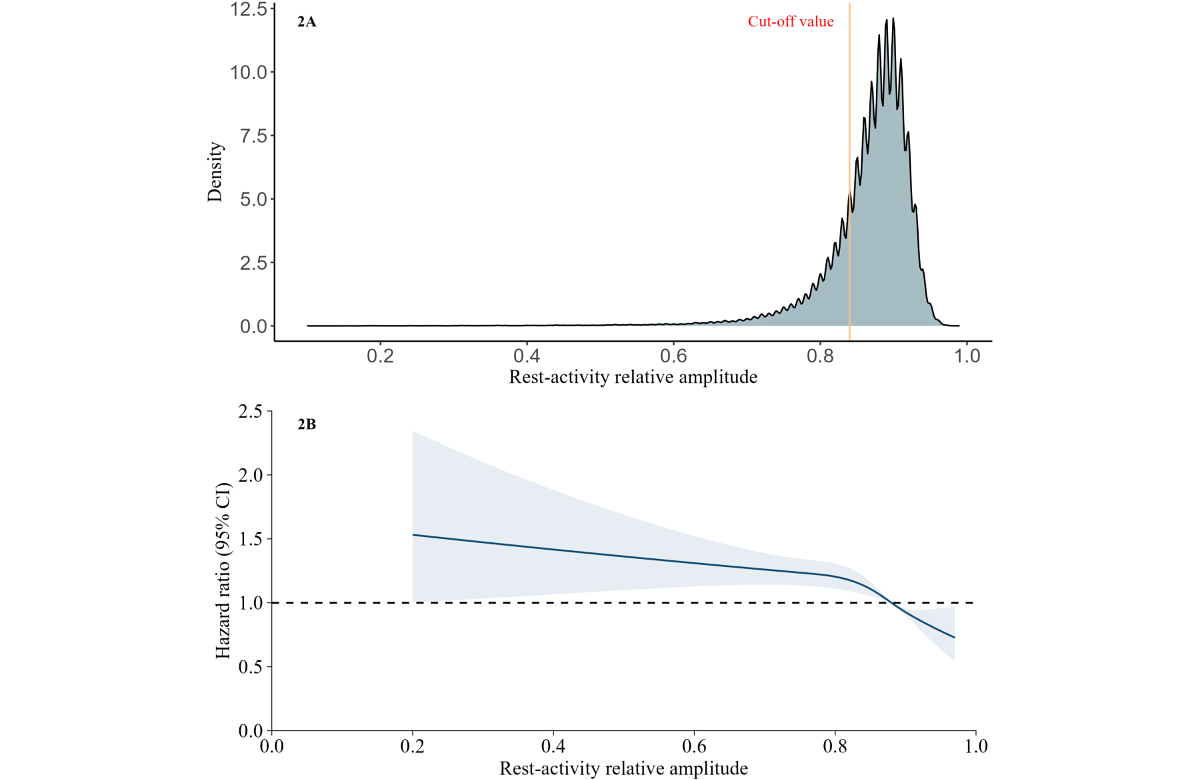
The distribution of continuous relative amplitude and the nonlinear association between rest-activity rhythm amplitude (RARA) and ischemic heart disease (IHD). In Figure 2A, RARA is defined as (M10 – L5) / (M10 + L5), whereas M10 indicates the average intensity of activity during the most active 10 hours within 24 hours and L5 indicates the average intensity of activity during the least active 5 hours within 24 hours, based on 1 week of wrist-worn accelerometer data from 84,095 participants. In Figure 2B, the model is fully adjusted. The reference value (hazard ratio [HR] 1; dashed horizontal line) was set at the median of continuous RARA (0.88). The shaded areas reflect the 95% CI for the HR.

The chosen focus of exposure was the relative amplitude of the rest-activity rhythm, a widely acknowledged circadian rhythm metric linked to various adverse health outcomes [7,9-12,24-28]. The R package “nparACT” [29] was used to calculate the parameters of M10 and L5. M10 is the average activity intensity during the most active 10 hours, whereas L5 is the average activity intensity during the least active 5 hours over 24 hours. RARA was calculated with the following equation: (M10 – L5) / (M10 + L5). RARA ranged from 0 to 1, with higher values indicating a robust rhythm. Subsequently, given the left-skewed distribution of RARA and evidence from previous studies suggesting threshold effects near the lowest quintile boundaries [7,30], we categorized the RARA into disrupted RARA (lowest quintile) and robust RARA (reference) groups. The descriptive distribution of the amplitude is provided in Figure 2A.

### SNP Selection

The optimal threshold for selecting SNPs as instrumental variables is typically set at the genome-wide statistical significance threshold of 5×10^−8^. However, in this case, only 4 SNPs met this stringent criterion. Consequently, to strike a balance between statistical significance and the inclusion of SNPs with potential biological significance, a more liberal cut-off of *P*<1×10^−5^ was used, following a previous study [31-33]. Identified SNPs were then clumped for linkage disequilibrium using the “clump_data” function from the TwoSampleMR package (R^2^=0.001 and >10 000 kb). In addition, an F-statistic of ≥10 was set to avoid the risk of weak instrument bias in MR analysis. Following harmonization of the exposure and outcome datasets and removal of palindromes, a final selection of 40 SNPs remained. Details of these SNPs are provided in Multimedia Appendix 1.

### Calculation of Polygenic Risk Score

The detailed information for the calculation process of the genetic risk score in the UK Biobank has been described in detail elsewhere [34]. Briefly, the polygenic risk scores for IHD (IHD-PRS) were generated using a Bayesian approach applied to meta-analyzed summary statistics from GWAS data. A subsequent principal component–based ancestry centering was applied to approximately center the score distributions on zero across all ancestries. Score distributions were also standardized to have approximately unit variance within ancestry groups, as determined by a geometric inference in principal component space.

### Outcome Definition

The diagnosis of IHD during the follow-up period was determined using the *ICD-10* codes (I20-25). This diagnosis, along with the date information, was derived from linked hospital admissions, death registries, and a subset of self-reported data.

Person-years of follow-up were calculated from the date the accelerometer was worn until the first instance of IHD, death, loss to follow-up, or the end of the follow-up period (England: October 31, 2022; Scotland: August 31, 2022; and Wales: May 31, 2022), whichever occurred first.

### Covariates Measurement

Referring to previous literature [9,10,12,26,27,35,36], potential confounding covariates include demographic, socioeconomic, health-related, and lifestyle factors, which were as follows: (1) demographics encompassed age (calculated from dates of birth and accelerometer wear date), sex (male or female), ethnicity (White, Black, Asian, or other); (2) socioeconomic status was reflected by educational attainment (higher education, any other qualification, and no qualification), region (England, Wales, or Scotland), and the Townsend Deprivation Index (TDI) [37]; (3) health-related factors included history of diabetes (yes or no), history of hypertension (yes or no), BMI level (normal or underweight [<25 kg/m^2^], overweight [≤25-<30 kg/m^2^], and obesity [≥30 kg/m^2^]), and the IHD-PRS [34] (categorized into “low” [lowest quartile], “mid” [second and third quartiles], and “high” [highest quartile]); and (4) lifestyle factors encompassed smoking status (never, previous, and current), alcohol consumption (not currently, up to twice per week, 3 or more times per week), and healthy diet (bad or good). Covariates were collected from the follow-up survey closest to the point at which the participant first wore the accelerometer. Season on the first day of accelerometer wear was included as a covariate to account for potential seasonal variations in activity patterns. Detailed covariate sources are provided in Table S2 in Multimedia Appendix 1.

### Statistical Analyses

#### Stage 1: Observational Analyses

The baseline characteristics were summarized as follows: continuous variables were presented as mean (SD), while categorical variables were represented as counts (N) and percentages (%). Cox proportional hazards regression was used to analyze the relationship between RARA and incident IHD, using time since accelerometer wear as the starting point for follow-up. Furthermore, interaction analysis was conducted by including an interaction term in the Cox proportional hazards models to examine the modifying effect of IHD-PRS. In Model 1, the crude models have no adjustment. It adjusted for demographics (age, sex, ethnicity, educational attainment, TDI, recruitment region, and season at start time). Model 2 further adjusted for lifestyle and health-related factors of alcohol status, smoking status, healthy diet, BMI, IHD-PRS, hypertension history, and diabetes history. Model 3 additionally added interaction terms between RARA and IHD-PRS. In addition, the joint effects between RARA and IHD-PRS on incident IHD were conducted by combining RARA and IHD-PRS into a single 6-category variable. The additive interaction effect—including relative excess risk due to interaction, attributable proportion, and synergy index—was calculated using the interactionR package [38], and CIs were obtained using the delta method. Subgroup analyses were stratified by age, sex, BMI, and IHD-PRS. The proportionality of hazards assumption was assessed using Schoenfeld residuals. While all models satisfied the assumption, we found a borderline result for exposure (*P*=.06; Figure S2 in Multimedia Appendix 1).

Sensitivity analyses were conducted by several approaches. First, a restricted cubic spline with the reference value of the median RARA was used to explore the nonlinear association. Second, individuals diagnosed with IHD within 1 year were excluded to minimize the potential for reverse causality. Third, the E-value [39], which is defined as the minimum strength of association that an unmeasured confounder would need to have with both the exposure and the outcome to fully explain away a specific exposure-outcome association, was computed to assess potential unmeasured confounders by the R package “EValue” [39]. Fourth, the analyses were restricted to White Europeans. Fifth, analyses were repeated by defining low-RARA as a cutoff of more than 2 SDs below the mean or using tertiles to transform RARA into a 3-category variable. Sixth, a subgroup sensitivity analysis was conducted to explore the association between RARA and IHD, which varied between acute and chronic conditions. Seventh, we added a sensitivity analysis using only hospital records and death registry data to define outcomes. Eighth, a sensitivity analysis, further adjusting for baseline characteristics such as chronic kidney disease and family history of cardiovascular disease, was conducted. Ninth, we censored data up to December 31, 2019 (to account for the start of the COVID-19 pandemic). Refer to Table S3 in Multimedia Appendix 1 for the sensitivity analyses results.

#### Stage 2: Mendelian Randomization

**Figure 3:**
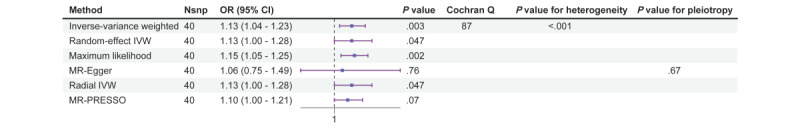
Mendelian randomization (MR) estimates of rest-activity rhythm amplitude (RARA) on ischemic heart disease (IHD). *P* value for heterogeneity was performed by fixed-effects IVW, and *P* value for pleiotropy was conducted by MR-Egger. IVW: inverse-variance weighted; MR: Mendelian randomization; MR-PRESSO: Mendelian randomization pleiotropy residual sum and outlier; Nsnp: number of single-nucleotide polymorphisms; OR: odds ratio.

The summary-level MR analysis was performed by the “TwoSampleMR” package [40]. Cochran Q statistic [41] was first computed to evaluate the heterogeneity induced by different genetic variants in the fixed-effects inverse-variance weighted (IVW) method. Then, horizontal pleiotropy was detected by the MR-Egger [42] method. The MR analysis was primarily performed by the IVW [43] approach. Several other well-established methods, including random-effects and radial IVW [44], MR-PRESSO [45], and maximum likelihood [46], were also performed for sensitivity analyses (Figure 3). The leave-one-SNP-out analysis was performed to detect the influence of individual genetic variants on the observed associations. Then we added a sensitivity analysis using different genome-wide significance thresholds (5×10^–5^, 5×10^–6^, 1×10^–6^, 5×10^–7^, 1×10^–7^, and 5×10^–8^). We further attempted to perform the above analysis after excluding SNPs that might be associated with potential confounders (*P*<1×10^–5^; PhenoScanner (version 2; Kamat MA) [47]).

The statistical analyses were completely conducted in R software (version 4.3.0; R Foundation) [48]. All statistical tests were 2-sided, and a *P*<.05 was considered significant.

### Ethical Considerations

The UK Biobank received ethics approval from the UK Biobank Research Ethics Committee (reference no 11/NW/0382) and was conducted in accordance with the Declaration of Helsinki. All participants signed informed consent allowing secondary analysis for health-related research purposes. All study data are deidentified. Participants were informed that they would not receive financial benefits from participation. We ensured that no identification of individuals in any images of the paper or additional material (ie, Multimedia Appendix 1) is possible. All participants provided written informed consent before participation.

## Results

### Baseline Characteristics of Observational Study Participants

In the UK Biobank observational study, a total of 84,095 individuals were eligible for analysis with valid parameters of RARA, no history of IHD, and other covariates. Among these participants, 3870 (4.60%) incident IHD events were detected during a median follow-up of 7.90 (IQR 7.33-8.41) years. Table 1 shows the baseline characteristics of the participants. The average age of participants was 61.54 (SD 7.84) years, 57.85% were women, and 43.98% reported having an education level of college and above. The time lag between the accelerometer measurement and the “closest” covariate assessment was 4.83 (SD 1.75) years and 5.12 (SD 2.07) years, respectively.

**Table 1:** Baseline characteristics of participants in the UK Biobank study.

Characteristic^a^		Participants (N=84,095)	Incident IHD^b^			*P* value	
			No (n=80,225)	Yes (n=3870)			
**Rest-activity rhythm amplitude (RARA), n (%)**							<.001
	Robust RARA	67,548 (80.32)	64,771 (80.74)	2777 (71.76)			
	Disrupted RARA	16,547 (19.68)	15,454 (19.26)	1093 (28.24)			
Age (years), mean (SD)		61.54 (7.84)	61.36 (7.84)	65.35 (6.78)	<.001		
**Sex, n (%)**							
	Female	48,646 (57.85)	47,168 (58.79)	1478 (38.19)	<.001		
	Male	35,449 (42.15)	33,057 (41.21)	2392 (61.81)			
**Ethnicity, n (%)**							
	White	81,557 (96.98)	77,804 (96.98)	3753 (96.98)	<.001		
	Asian	920 (1.09)	857 (1.07)	63 (1.63)			
	Black	708 (0.84)	685 (0.85)	23 (0.59)			
	Other	910 (1.08)	879 (1.10)	31 (0.80)			
**Educational attainment, n (%)**							<.001
	No qualification	6541 (7.78)	6055 (7.55)	486 (12.56)			
	Any other qualification	40,568 (48.24)	38,634 (48.16)	1934 (49.97)			
	College or higher	36,986 (43.98)	35,536 (44.30)	1450 (37.47)			
TDI^c^, mean (SD)		–1.74 (2.81)	–1.75 (2.81)	–1.64 (2.85)	.03		
**Alcohol status, n (%)**							<.001
	Not current	4921 (5.85)	4632 (5.77)	289 (7.47)			
	2 or fewer times a week	38,997 (46.37)	37,238 (46.42)	1759 (45.45)			
	3 or more times a week	40,177 (47.78)	38,355 (47.81)	1822 (47.08)			
**Smoke status, n (%)**							<.001
	Never	49,059 (58.34)	47,160 (58.78)	1899 (49.07)			
	Previous	29,784 (35.42)	28,141 (35.08)	1643 (42.45)			
	Current	5252 (6.25)	4924 (6.14)	328 (8.48)			
**Healthy diet^d^, n (%)**							<.001
	Bad	35,046 (41.67)	33,284 (41.49)	1762 (45.53)			
	Good	49,049 (58.33)	46,941 (58.51)	2108 (54.47)			
**BMI, n (%)**							<.001
	Normal or underweight	34,213 (40.68)	33,109 (41.27)	1104 (28.53)			
	Obesity	15,642 (18.60)	14,603 (18.20)	1039 (26.85)			
	Overweight	34,240 (40.72)	32,513 (40.53)	1727 (44.63)			
**IHD-PRS^e^, n (%)**							<.001
	Low	22,476 (26.73)	21,774 (27.14)	702 (18.14)			
	Intermediate	42,320 (50.32)	40,409 (50.37)	1911 (49.38)			
	High	19,299 (22.95)	18,042 (22.49)	1257 (32.48)			
**Season at start time, n (%)**							.90
	Spring	19,116 (22.73)	18,237 (22.73)	879 (22.71)			
	Summer	21,906 (26.05)	20,903 (26.06)	1003 (25.92)			
	Autumn	25,122 (29.87)	23,947 (29.85)	1175 (30.36)			
	Winter	17,951 (21.35)	17,138 (21.36)	813 (21.01)			
**Recruitment region, n (%)**							<.001
	England	75,457 (89.73)	71,807 (89.51)	3650 (94.32)			
	Wales	3172 (3.77)	3126 (3.90)	46 (1.19)			
	Scotland	5466 (6.50)	5292 (6.60)	174 (4.50)			
**Hypertension history, n (%)**							<.001
	No	66,380 (78.93)	63,926 (79.68)	2454 (63.41)			
	Yes	17,715 (21.07)	16,299 (20.32)	1416 (36.59)			
**Diabetes history, n (%)**							<.001
	No	81,139 (96.48)	77,600 (96.73)	3539 (91.45)			
	Yes	2956 (3.52)	2625 (3.27)	331 (8.55)			

^a^Data are presented as the mean (SD) for continuous variables and number (%) for categorical variables.

^b^IHD: ischemic heart disease.

^c^TDI: Townsend Deprivation Index.

^d^According to the Dietary Guidelines for Americans (DGA) recommendation, a healthy eating pattern includes a specific amount of consumption for a variety of fruits, vegetables, fish, lean proteins (red and processed meat), whole and refined grains, and low-fat dairy products. The healthy diet category was defined to include at least 4 of the following 7 food groups: (1) fruits: ≥3 servings/day, (2) vegetables: ≥ 3 servings/day, (3) fish: ≥2 servings/week, (4) processed meats: ≤1 serving/week, (5) unprocessed red meats: ≤1.5 servings/week, (6) whole grains: ≥3 servings/day, and (7) refined grains: ≤1.5 servings/day

^e^IHD-PRS: polygenic risk scores for ischemic heart disease.

### Relations Among RARA, IHD-PRS, and IHD in Observational Study

The associations between RARA and incident IHD are provided in Table 2. RARA disturbance showed an increased risk of IHD in all models. Participants with a disrupted RARA had a 1.20-fold increase in incident IHD compared with those with a robust RARA in the fully adjusted model (hazard ratio [HR] 1.20, 95% CI 1.12-1.30). The restricted cubic spline analysis of continuous RARA showed a consistent trend (Figure 2B). In addition, subgroup analyses stratified by age, sex, BMI, and IHD-PRS categories revealed similar results (Figure S3 in Multimedia Appendix 1).

No significant modification effects of IHD-PRS were found (HR 0.92, 95% CI 0.76-1.11; *P*=.39 and HR 0.91, 95% CI 0.74-1.12; *P*=.37, respectively), suggesting that RARA is an intervenable risk factor independent of genetic predisposition. Moreover, the joint effect combining RARA and IHD-PRS is provided in Figure S4 and Table S5 in Multimedia Appendix 1. Unsurprisingly, participants with robust RARA and low IHD-PRS have the lowest risk for incident IHD, and the 2 factors independently influence the occurrence of IHD, whether the interaction is on a multiplicative or additive scale.

**Table 2:** Associations of rest-activity rhythm amplitude (RARA) and polygenic risk scores for ischemic heart disease (IHD-PRS) with ischemic heart disease (IHD) in the UK Biobank study.

Analysis model^a^	HR^b^ (95% CI)		E-value		
Robust RARA^c^ (ref)		1		—^d^	
Disrupted RARA		—		—	
Crude model		1.66^e^ (1.54-1.78)		2.71	
Model 1		1.43^e^ (1.34-1.54)		2.21	
Model 2 (fully adjusted model)		1.20^e^ (1.12-1.30)		1.69	
Low (ref)		1		—	
Intermediate		1.46^e^ (1.34-1.59)		2.30	
High		2.17^e^ (1.98-2.38)		3.76	
Model 3 (interaction model)		—		—	
Multiplicative interaction scale		—		—	
Interaction term of disrupted RARA and intermediate IHD-PRS^f^		0.92 (0.76-1.11)		—	
Interaction term of disrupted RARA and high IHD-PRS		0.91 (0.74-1.12)		—	
Additive interaction scale		—		—	
High RARA and low IHD-PRS (ref)		1		—	
High RARA and intermediate IHD-PRS		1.50^e^ (1.35-1.66)		2.37	
Disrupted RARA and low IHD-PRS		1.29^e^ (1.10-1.52)		1.90	
Disrupted RARA and intermediate IHD-PRS		1.78^e^ (1.57-2.01)		2.96	
High RARA and high IHD-PRS		2.23^e^ (2.00-2.49)		3.89	
Disrupted RARA and high IHD-PRS		2.63^e^ (2.29-3.02)		4.70	

^a^Crude model: no adjustment. Model 1: adjusted for age, sex, ethnicity, educational attainment, TDI, recruitment region, and season at start time. Model 2 (fully adjusted model): additionally, adjusted for alcohol status, smoking status, healthy diet, BMI, IHD-PRS, hypertension history, and diabetes history. Model 3 (interaction model): interaction terms of RARA with IHD-PRS were added.

^b^HR: hazard ratio.

^c^RARA: rest-activity rhythm amplitude.

^d^Not applicable.

^e^*P*<.001.

^f^IHD-PRS: polygenic risk scores for ischemic heart disease.

### Causality Among RARA and IHD in Mendelian Randomization

The results of the MR analysis to investigate the causality of RARA and IHD are provided in Figure 3 and Table 3. The intercept term from the MR-Egger regression analysis suggests the absence of clear directional pleiotropy (intercept: 0.002; *P*=.67). IVW methods detected heterogeneity in the SNPs (*P*<.001). The random-effects IVW method showed that genetically determined disrupted RARA is associated with an increased risk of IHD (odds ratio [OR] 1.13, 95% CI 1.00-1.28; *P*=.047), suggesting a likely causal relationship. Table 3 demonstrates that while relaxing the *P-*value threshold increases statistical power by incorporating more SNPs. the results at the 5×10⁻⁸ threshold lacked statistical significance due to insufficient statistical power resulting from too few SNPs (OR 1.02, 95% CI 0.84-1.24; *P*=.88). However, the point estimates suggested an adverse effect, and the direction of the causal effect remains consistent across thresholds. Importantly, the strength of the 40 selected SNPs was robust, with F-statistics ranging from 19.55 to 60.63 (median 21.61), all well above the conventional threshold of 10, suggesting that weak instrument bias is unlikely. The results of the sensitivity analysis are provided in Figures S5-S8 in Multimedia Appendix 1, which were also consistent with the analyses, and details of SNPs are provided in Table S4 in Multimedia Appendix 1. Results were also consistent for analyses that further excluded 6 confounding-related SNPs (OR 1.16, 95% CI 1.01-1.33; IVW method).

**Table 3:** Results of different *P* value thresholds for Mendelian randomization.

*P* threshold	OR^a^ (95% CI)	*P* value	SNP^b^
5×10^–5^	1.09 (1.04-1.15)	.001	128
1×10^–5^	1.13 (1.00-1.28)	.047	40
5×10^–6^	1.12 (1.01-1.23)	.03	27
1×10^–6^	1.15 (1.00-1.32)	.049	11
5×10^–7^	1.16 (1.00-1.35)	.049	9
1×10^–7^	1.02 (0.84-1.23)	.88	4
5×10^–8^	1.02 (0.84-1.23)	.88	4

^a^OR: odds ratio.

^b^SNP: single-nucleotide polymorphism.

## Discussion

### Principal Findings

To the best of our knowledge, this is the first study to comprehensively investigate the relationship between RARA, genetic susceptibility, and IHD in a large general population, combining evidence from observational studies and MR analyses. Based on approximately 85,000 participants in the UK Biobank, we found that RARA disturbances were associated with an increased risk of IHD, and there is no evidence that genetic predisposition modifies such an association. Furthermore, we identified a potential causal relationship by using MR methods. Our triangulation results provide suggestive evidence supporting the essential role of RARA by demonstrating its potential independent causality for the future risk of IHD. Although the magnitude of the effect was modest and may limit its predictive value for individual risk stratification, even modest population-level risks can translate into a meaningful public health impact when exposure is highly prevalent, as with air pollution.

### Comparisons With Previous Studies of RARA

Our study revealed a heightened risk of incident IHD associated with RARA disturbances. This finding was supported by 2 studies with small samples of special populations and a cross-sectional study. The Osteoporotic Fractures in Men study [14] found an association between RARA and increased risk of coronary heart disease events in 2968 men aged 76.3 years (HR 1.39, 95% CI 1.02-1.91; the greatest risk among men in highest quartile compared with the lowest quartile). A higher association was observed in the 3147-person diabetic population [12] (HR 2.45, 95% CI 1.73-3.49), which is similar to the cross-sectional study. The difference between these studies may be attributable to the different techniques used to measure RARA, in addition to differences in the study population (age, sex, geography, baseline comorbidity) and methods used to ascertain the outcome. In addition, it is also possible that our model estimates the direct effect of RARA disturbances, as we adjusted for BMI and diabetes within the model, which may be mediators in the causal pathway. Using a general and much larger sample size population (N=84,095), our study provided convincing evidence for the link between RARA and the incidence of IHD. Furthermore, the E-value for the fully adjusted model was 1.69, suggesting that unmeasured confounders of moderate strength could potentially explain the results. It is worth noting that this association was statistically significant in both the chronic IHD and acute IHD populations. It is clinically relevant to include monitoring of RARA in the screening of populations at risk for IHD, regardless of acute or chronic IHD.

### The Modifying Effect of Genetic Predisposition

Contrary to the widely held viewpoints in previous studies suggesting that genetic predisposition typically modifies the effects of environmental factors and health behaviors on IHD [15], our investigation identifies RARA as a risk factor for IHD that is independent of genetic predisposition. This finding is supported by several previous studies that investigated the relationship between RARA, genetic predisposition, and diabetes [9] or atrial fibrillation [10], suggesting that attention should be accorded to individuals’ RARA, irrespective of their genetic susceptibility. In addition, the screening of SNPs in the process of constructing genetic risk scores and the sample size of the study may also contribute to the nonsignificance of the effect modification.

### Causality Between RARA and IHD

Furthermore, in contrast to the preceding studies that concentrated solely on exploring epidemiological associations, incorporating MR in our study provides enhanced confidence in the potential causal nature of the relationship between RARA and IHD, thereby complementing and extending the understanding of its potential as a crucial intervention target for the development of preventive and therapeutic strategies for IHD.

Although MR analysis can test causal effects, it needs to satisfy 3 hypothesis assumptions. First, the relevance assumption, indicating that SNPs should be strongly correlated with exposure. To meet this assumption, we selected the F-statistic ≥10 to avoid the risk of weak instrument bias in our MR analysis. Second, the independence assumption, meaning that SNPs share no unmeasured confounders with the outcome. For this, we performed a sensitivity analysis excluding the 6 potential SNPs. The results remained robust after excluding related SNPs. Third, the exclusion restriction assumption implies that SNPs should not influence the outcome except through the exposure of interest. Our sensitivity analysis, such as MR-Egger, revealed no evidence of horizontal pleiotropy in our MR study. Although the MR analysis detected significant heterogeneity, no evidence of directional (unbalanced) pleiotropy was found based on the MR-Egger test. This pattern suggests that the observed heterogeneity may reflect genuine biological variability in the causal pathways rather than systematic bias. It may simply be that different variants affect the exposure in different ways, but there are no invalid SNPs [49]. Another possible reason is balanced pleiotropy. Although some pleiotropic pathways may be present, their effects appear to be bidirectional and offsetting rather than consistently biased in one direction. Consequently, they contribute to heterogeneity and inflate the variance of our estimates without introducing systematic bias into the causal effect [50]. As the core assumptions remain unviolated, the estimates are still valid. The random-effects IVW method accommodates such heterogeneity, and consistent results further validate the robustness of the estimates. What is more, the limited sample size of the GWAS restricted our ability to identify genetic instruments at the genome-wide significance threshold (*P*<5×10⁻⁸), yielding only 4 SNPs that did not demonstrate significant associations in the subsequent MR analysis. To increase statistical power, we adopted a series of relaxed thresholds while ensuring adequate instrumental variable strength (F>10), which revealed significant causal relationships. Although the strength of the 40 selected SNPs was robust, with F-statistics ranging from 19.55 to 60.63, well above the conventional threshold of 10, we acknowledge that this approach may still introduce slight uncertainty; future MR studies based on larger GWAS datasets are needed to confirm these findings. It is worth noting that the MR estimate (OR 1.13, 95% CI 1.00-1.28) is very close to the observational study (HR 1.20, 95% CI 1.12-1.30). The similarity between the MR and observational estimates may be partially driven by the partial sample overlap between the exposure GWAS (UK Biobank) and the observational cohort (UK Biobank). Overall, our MR study is valid and credible, and supports a potential causal relationship between RARA and IHD.

### The Potential Mechanisms by Which RARA Contributes to Incident IHD

There are several potential mechanisms through which RARA may lead to a higher incidence of IHD. First, for possible direct regulation pathways, circadian machinery plays a pivotal role in governing the expression of cardiovascular function genes and modulating the levels of cardiovascular proteins [51,52]. In addition, it influences the levels of neurohormones responsible for regulating cardiovascular function, such as atrial natriuretic peptide [53]. Second, for possible indirect regulation mechanisms, RARA disturbances are considered potential physiological correlates of frailty development, and frail individuals have a reduced ability to recover from stressful events, making them more susceptible to common chronic diseases [36]. Third, more microscopically, previous studies have linked RARA disturbances to changes in metabolism [8] and inflammatory markers [24]. RARA disturbances may include a sedentary lifestyle (ie, low M10 contributing to RARA disturbances) and poor sleep (ie, higher L5 contributing to RARA disturbances), which in turn affects metabolism, inflammatory cascades, and the autonomic nervous system. Given the key roles of metabolic function and inflammation in IHD [54-57], circadian disruptions may increase IHD risk by negatively impacting metabolism and exacerbating inflammation. It is worth noting that future studies should focus on elucidating the multiple mechanisms linking RARA with incident IHD.

### Strengths and Limitations

Our investigation has some distinguishing strengths. First, the use of a triangulation approach combining longitudinal cohort observational studies and MR studies lends credibility to our findings because it is less susceptible to bias from confounding and reverse causation. However, since the observational study uses the UK Biobank and the exposure GWAS also uses the UK Biobank, the observational cohort is effectively a subset of the exposure GWAS cohort, which limits the independence of the 2 lines of evidence, particularly regarding exposure measurement error, even though the outcome samples are independent. Second, in a large sample size of 84,095 disease-free general population with long duration of follow-up, objectively measuring 24-hour accelerometer data and *ICD-10* diagnosis allowed us to characterize a more realistic relationship with less misclassification and recall bias. Finally, our MR analysis was based on a GWAS with a large sample size, and its results appeared robust to different sensitivity analyses.

There are still some limitations to consider. First, although the sample includes multiethnic and general people in an observational study, the proportion of White people is 96.68% and participants has a mean age of 61.54 (SD 7.84) years, and there is still a lack of diversity. Future studies need to examine the relationship in younger and multiethnic groups to identify the optimal timing of intervention. Second, given that the UK Biobank is composed of volunteer participants, this could result in a sample that leans toward individuals who are healthier than the general population. Third, certain covariates, such as lifestyle factors, were not gathered at the baseline of this study (accelerometer measured), and instead relied on physical follow-up to the UK Biobank assessment centers. However, we have selected the follow-up closest to the measurement time as the variable source to minimize potential bias. In addition, previous literature [7,58] has found that participants’ covariates are stable across multiple follow-up visits, so the bias in the study results could be negligible. Fourth, despite ensuring the strength of the instrumental variables, the relaxed threshold may still affect the robustness of the causal evidence. Future studies could validate this causality further based on GWAS with larger samples. Fifth, partial overlap in the sample between the exposure GWAS and the observational cohort could introduce the “winner’s curse,” which could bias the 2-sample MR estimates toward the observational associations. This overlap could inflate the apparent causal effect size. In addition, while the UK Biobank and FinnGen are independent cohorts with distinct geographical recruitment regions (the United Kingdom and Finland, respectively), we acknowledge the possibility of minimal participant overlap (eg, due to cross-border migration during recruitment periods). Future MR studies using fully independent samples are needed to validate our findings.

### Clinical Implications

This research offers several important implications for mitigating the risk of IHD. The study hints at potential therapeutic avenues such as lifestyle modifications (eg, consistent sleep schedules and regular physical activity), bright light therapy, chronotherapeutic interventions [59], or melatonin supplementation [60], which may target the improvement of rest-activity rhythm to reduce the risk of IHD among affected individuals.

### Conclusion

Disrupted RARA was associated with an increased risk of IHD through a triangulation approach. It should be noted that the causal finding was sensitive to the statistical threshold used for instrument selection. This relationship is independent of genetic predisposition, highlighting the significance of RARA improvement for IHD prevention in the whole population, particularly the older population.

## Appendix

Multimedia Appendix 1 Additional methods, figures, and tables.

## Abbreviations

GWAS: genome-wide association study
HR: hazard ratio
ICD-10: International Statistical Classification of Diseases, Tenth Revision
IHD: ischemic heart disease
IHD-PRS: polygenic risk scores for IHD
IVW: inverse-variance weighted
MR: Mendelian randomization
OR: odds ratio
RARA: rest-activity rhythm amplitude
SNP: single-nucleotide polymorphism
TDI: Townsend Deprivation Index

## Data Availability

Individual-level data are available via application to the UK Biobank. Data used for MR were obtained from publicly available genome-wide association studies.
