# Supplementary material for: Accelerometer-Derived Rest-Activity Rhythm Amplitude, Genetic Predisposition, and the Risk of Ischemic Heart Disease: Observational and Mendelian Randomization Study
Source: J Med Internet Res. 2025 Dec 15;27:e79301. doi: 10.2196/79301 (PMC12750073; doi:10.2196/79301)
Supplement: Multimedia Appendix 1 [file jmir_v27i1e79301_app1.docx]

**Additional Ma****terials**

Contents

[Methods 3](#_Toc212317537)

[Figures 4](#_Toc212317538)

[Figure S1. Flowchart of participant enrolment 4](#_Toc212317539)

[Figure S2. Scaled Schoenfeld residual plots for the proportionality of hazards assumption of the fully-adjusted model. 5](#_Toc212317540)

[Figure S3. Subgroup Analysis by IHD-PRS, Sex, Age, and BMI. 6](#_Toc212317541)

[Figure S4. Joint effects of RARA and IHD-PRS on the incidence of IHD. 7](#_Toc212317542)

[Figure S5. Scatter plots for summary-level Mendelian randomization. 8](#_Toc212317543)

[Figure S6. Forest plot of the individual and combined effect of RARA on IHD. 9](#_Toc212317544)

[Figure S7. The leave-one-out estimate of RARA on IHD. 10](#_Toc212317545)

[Figure S8. Funnel plot for RARA on IHD analysis. 11](#_Toc212317546)

[Tables 12](#_Toc212317547)

[Table S1. Detailed information on the GWAS data analyzed in this study. 12](#_Toc212317548)

[Table S2. The information source of covariates. 12](#_Toc212317549)

[Table S3. Sensitivity analyses for associations of RARA with risks of incident IHD. 13](#_Toc212317550)

[Table S4. Characteristics of genetic variants used to estimate the effect of RARA on IHD. 15](#_Toc212317551)

[Table S5. The additive interaction of RARA and IHD-PRS with IHD in the UK Biobank Study. 17](#_Toc212317552)

# Methods

Between February 2013 and December 2015, 236,519 UK Biobank participants were enlisted for the accelerometer study. Consent to participate was received from a total of 106,053 participants, who were then supplied with an Axivity AX3 wrist-mounted accelerometer. This device was programmed to start functioning at 10 a.m. on the second business day subsequent to its postal dispatch and to record triaxial acceleration data over a span of seven days at a frequency of 100 Hz with a dynamic field of ± 8 gravity (9.8 m/s^2^). The participants were guided to wear the device on their dominant wrist nonstop for seven days, without any change in their usual activities. Upon completing the designated seven-day monitoring period, participants were requested to return the device to the coordinating institutions via mail using a prepaid envelope. As a result, a collection of 103,712 raw accelerometer datasets was gathered for subsequent data analysis.

In summary, the UK Biobank accelerometer working group pinpointed stationary intervals in ten-second windows when all three axes had a standard deviation less than 13.0 mg. These static periods were used to fine-tune the gain and offset for each axis (nine variables) to model a gravity sphere unit through the ordinary least squares linear regression. In cases where adequate data for calibration were missing for any participant (for instance, any of the three sensor axes did not garner values outside a +- 300 mg range), the previously established calibration coefficients were used from another participant who wore the same device previously (or next, if former data were unavailable). Values that surged over the sensor's dynamic range of +-8g, resulting in "clipping," were flagged pre- and post-calibration. Instances of recording errors and 'interrupts', which might have occurred when participants attempted to connect their accelerometer device to a computer, were also logged. The admissible data were then resampled to 100 Hz using linear interpolation. More specifics regarding the processing and analysis of the data can be found in a separate publication.

Based on the data quality metrics provided by the UK Biobank accelerometer working group, the exclusion criteria are as follows: 1) those data flagged by UK Biobank as being unreliable due to unexpectedly small or large size (Field ID: 90002; n=4694); 2) those with accelerometry data for less than 72 h or did not provide data for all 1-h periods within a 24-h cycle during the 7-day data collection (Field ID: 90015; n=4474); 3) those data identified by UK Biobank as not well-calibrated (Field ID: 90016; n=3); 4) those data were recalibrated using the previous accelerometer record from the same device worn by a different participant (Field ID: 90017; n=154); 5) those data with a non-zero count of interrupted recording periods (Field ID: 90180; n=1653); 6) those data with more than 767.5 (Q3 + 1·5 × IQR) data recording errors (Field ID: 90182; n=85).

# Figures

## Figure S1. Flowchart of participant enrollment.


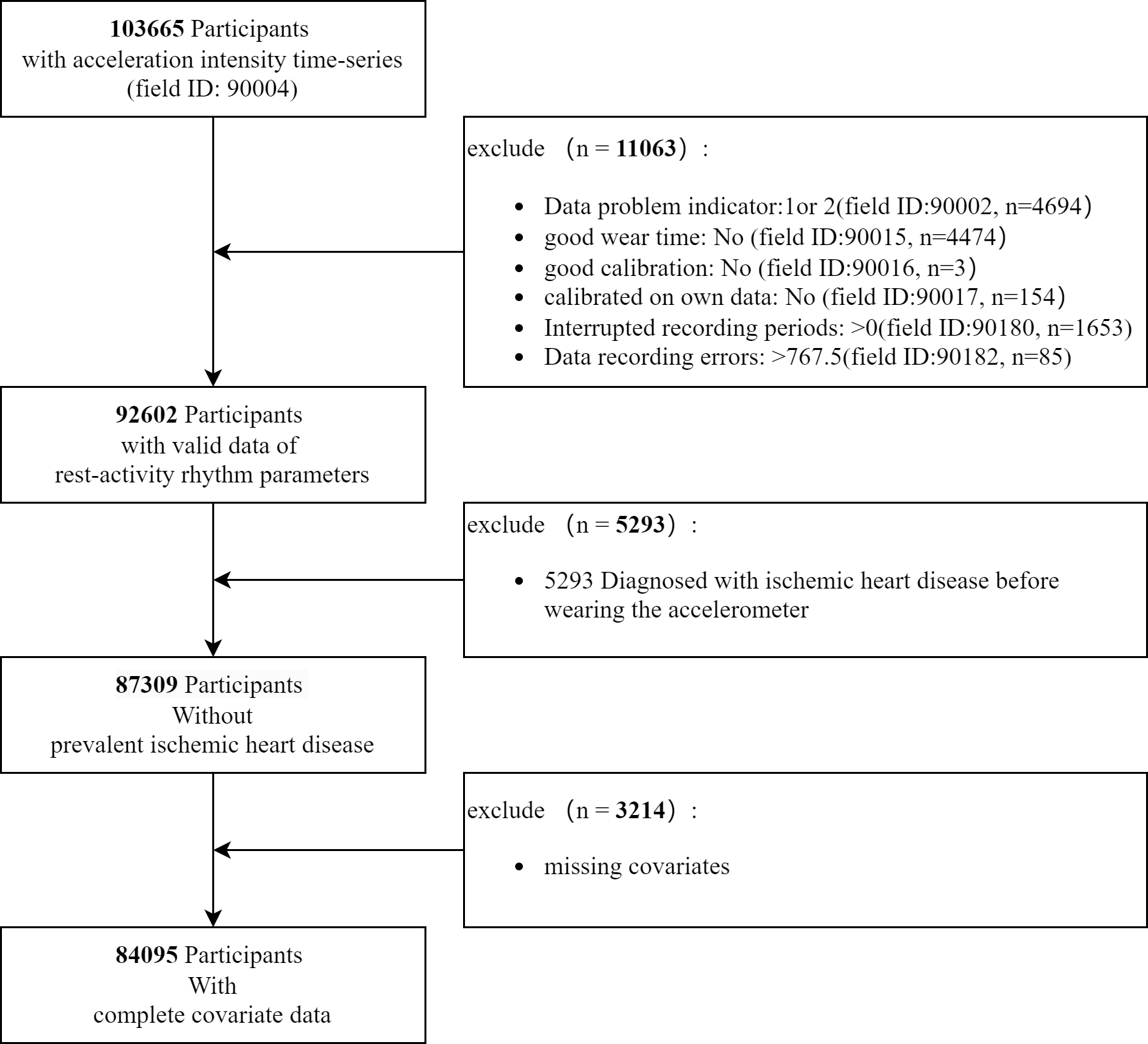


## Figure S2. Scaled Schoenfeld residual plots for the proportionality of hazards assumption of the fully-adjusted model.


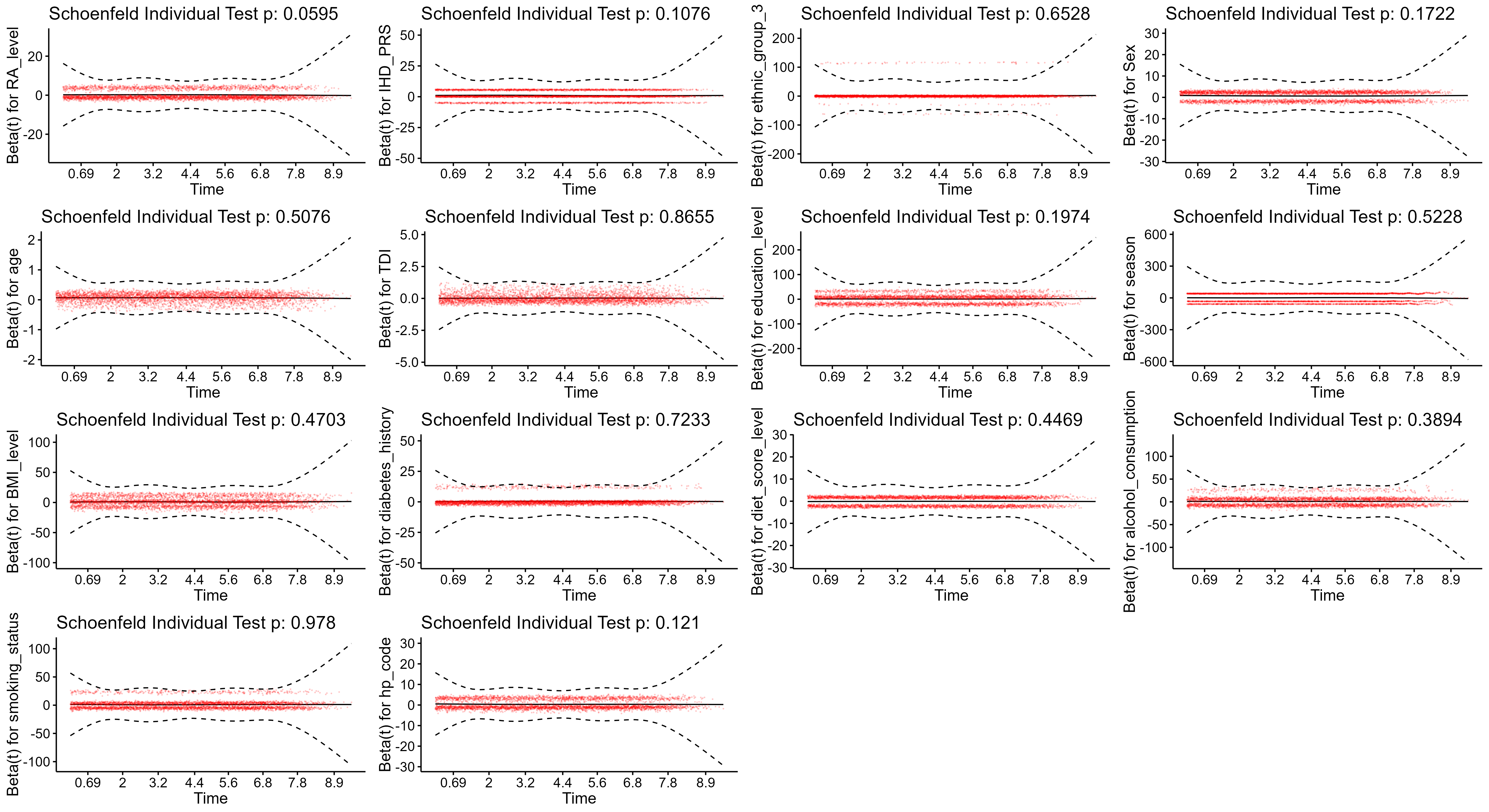


## Figure S3. Subgroup analysis by IHD-PRS, Sex, Age, and BMI.


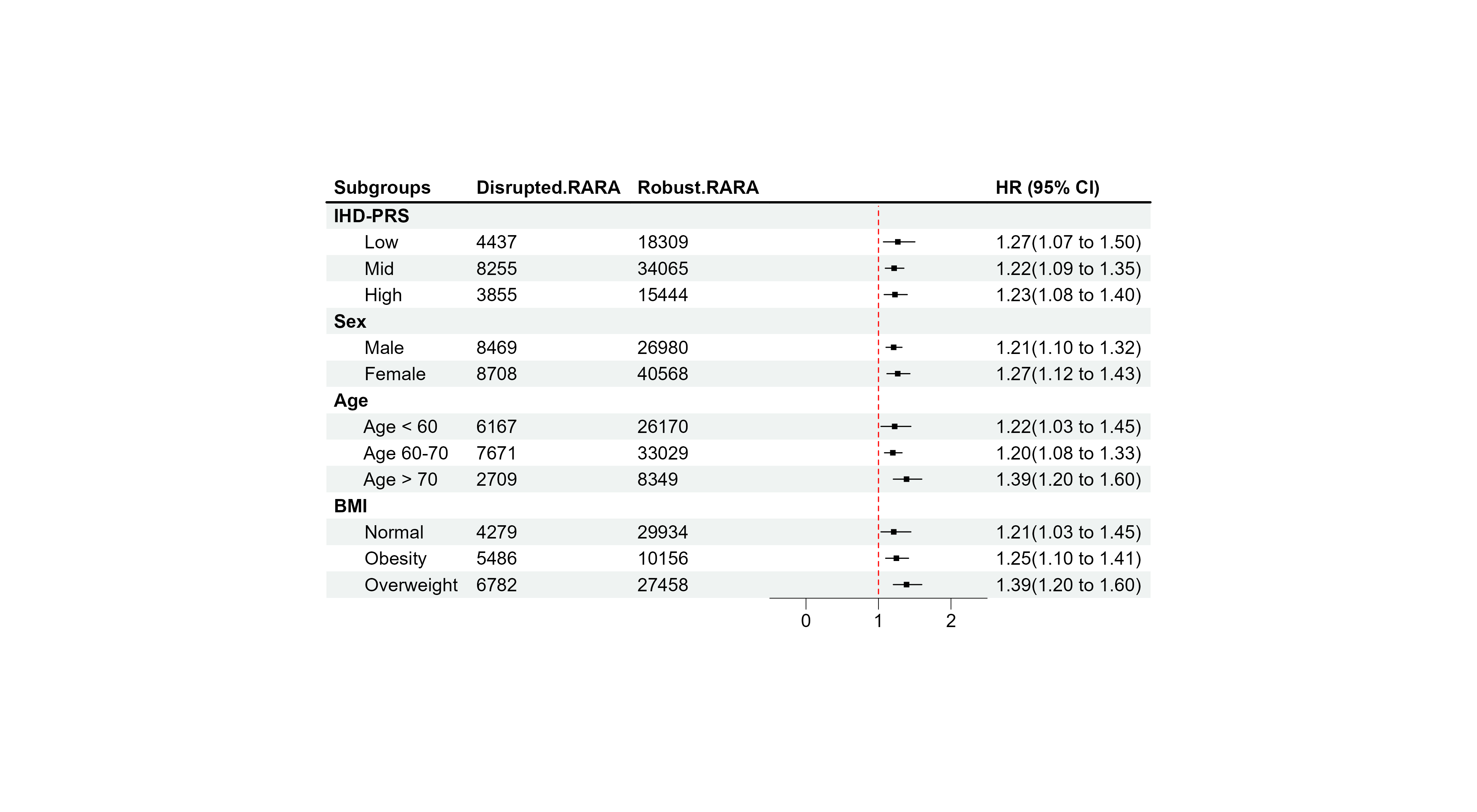


IHD-PRS (categorized into “low” [lowest quartile], “mid” [second and third quartiles], and “high” [highest quartile]); BMI (normal (<25 kg/m2), overweight (≤25-<30 kg/m2), and obesity [≥30 kg/m2]).

## Figure S4. Joint effects of RARA and IHD-PRS on the incidence of IHD.





## Figure S5. Scatter plots for summary-level Mendelian randomization.


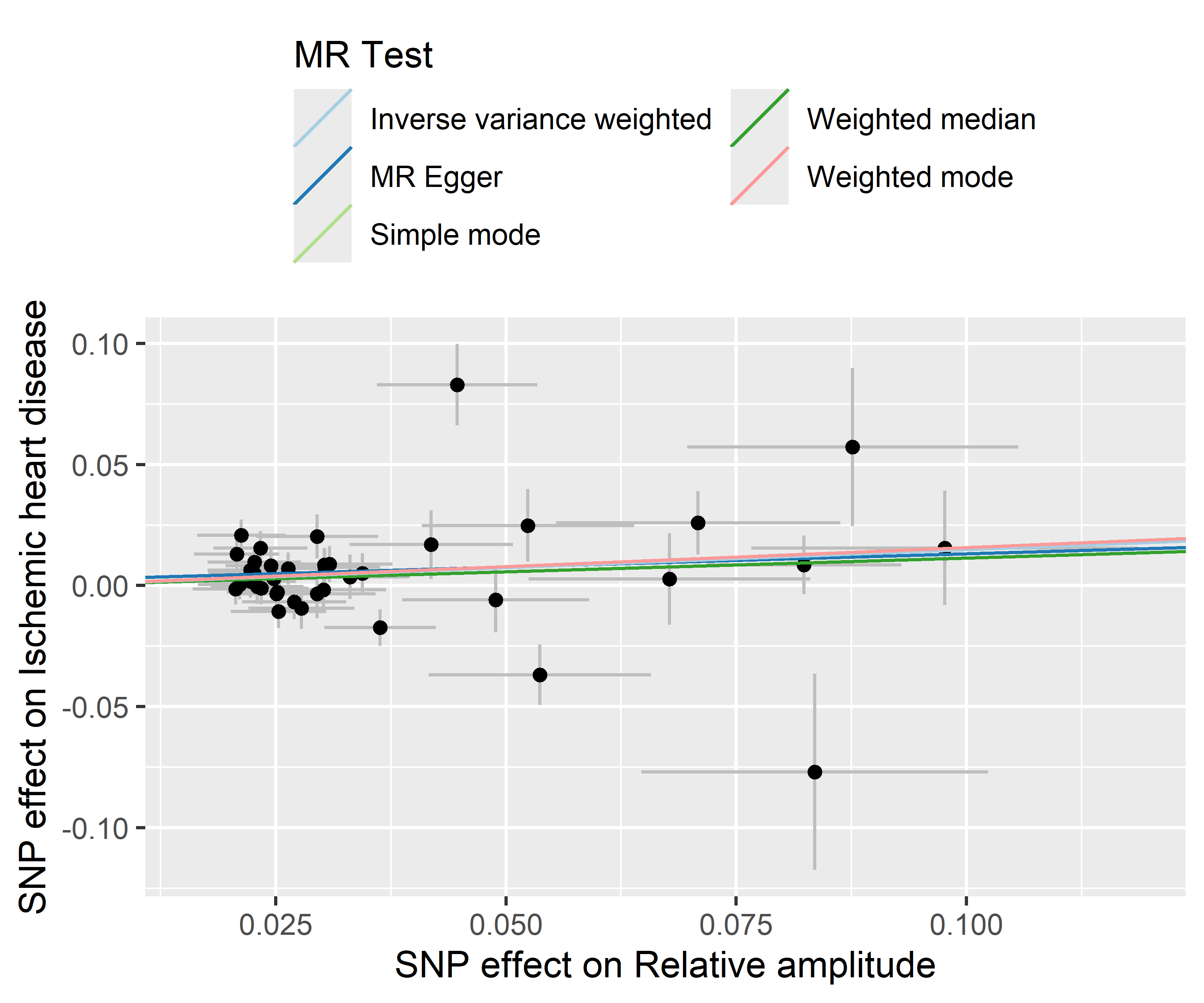


## Figure S6. Forest plot of the individual and combined effect of RARA on IHD. Data are presented as β with the 95% CI.


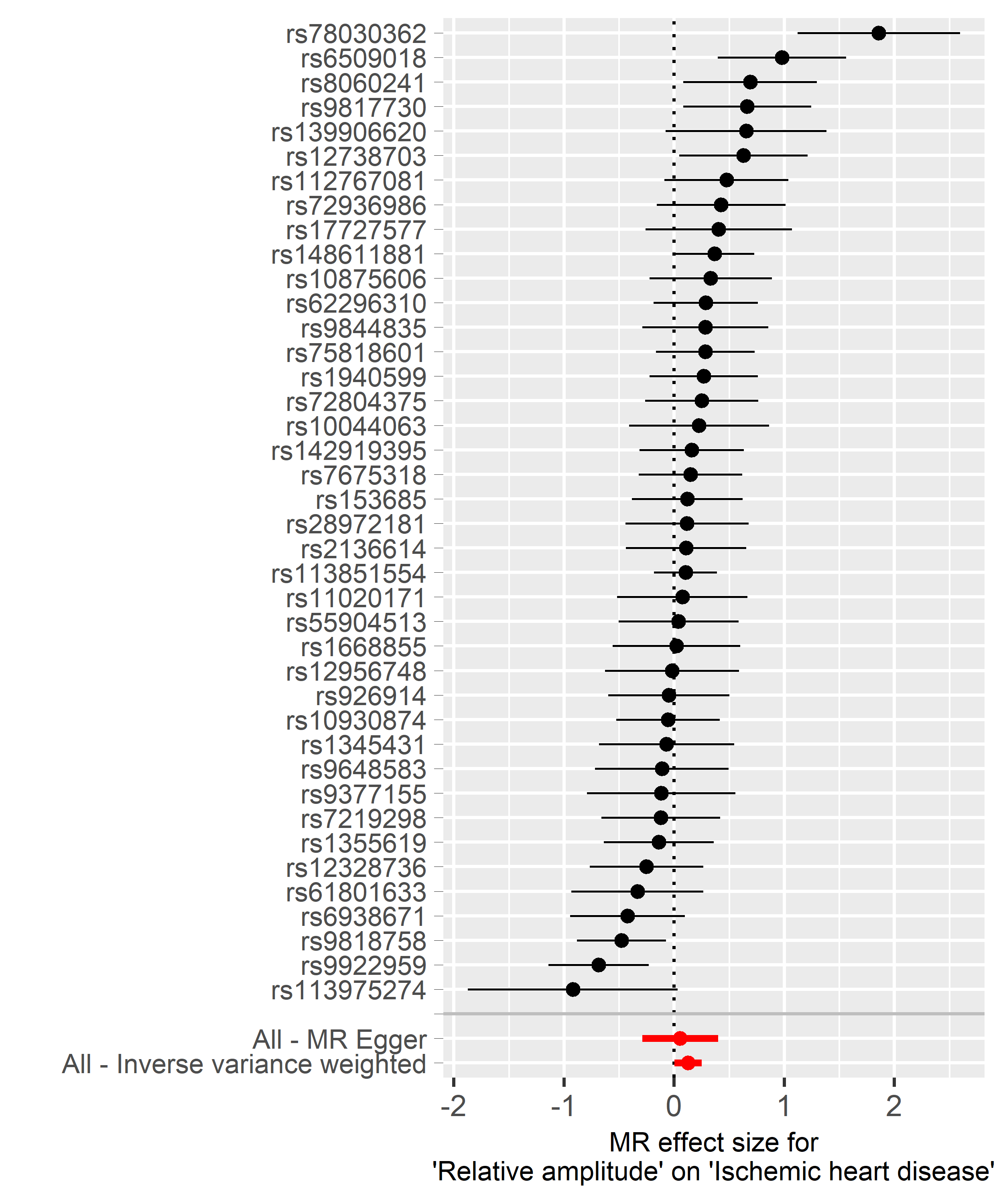


## Figure S7. The leave-one-out estimate of RARA on IHD. Data are presented as β with the 95% CI.


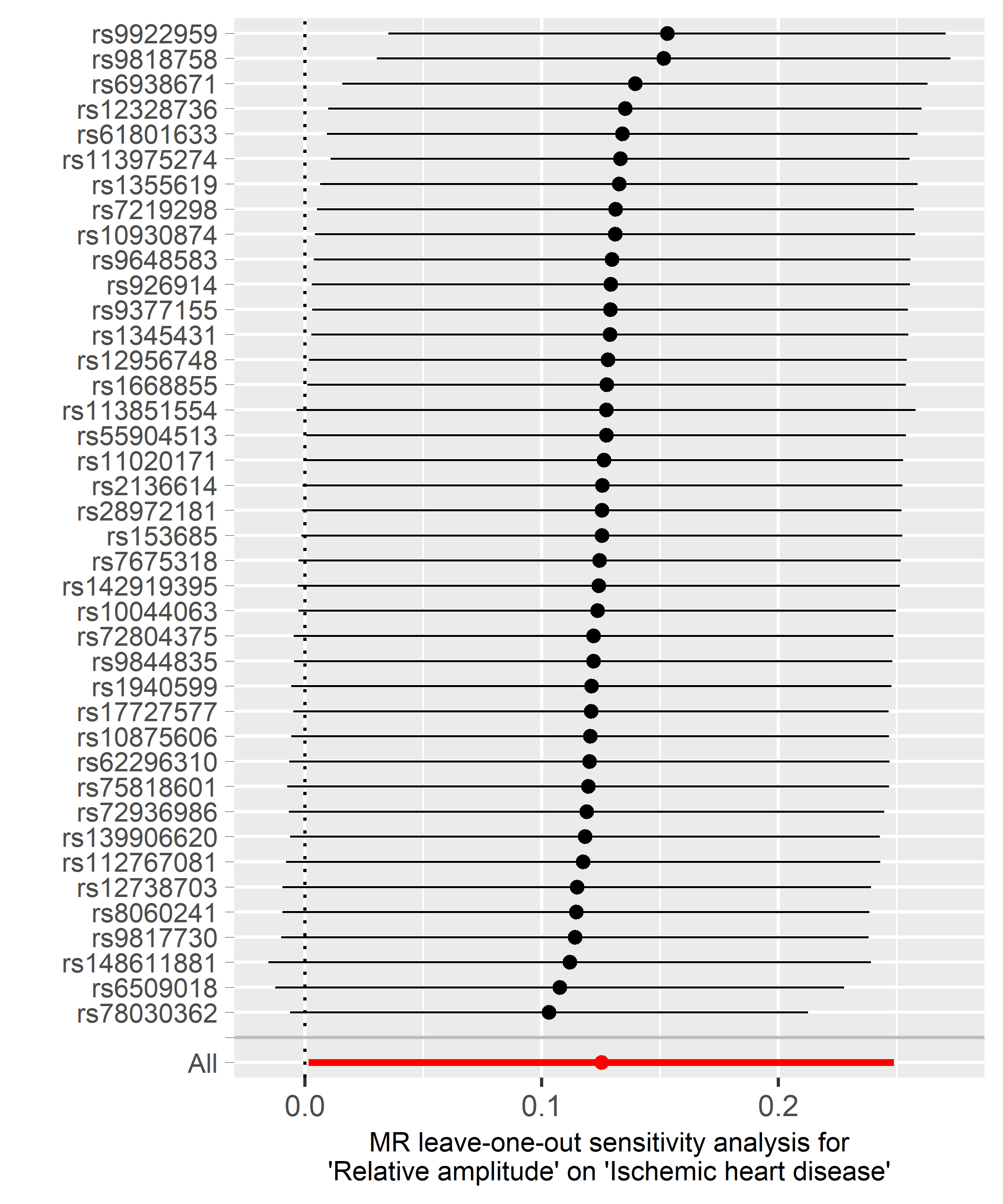


## Figure S8. Funnel plot for RARA on IHD analysis.


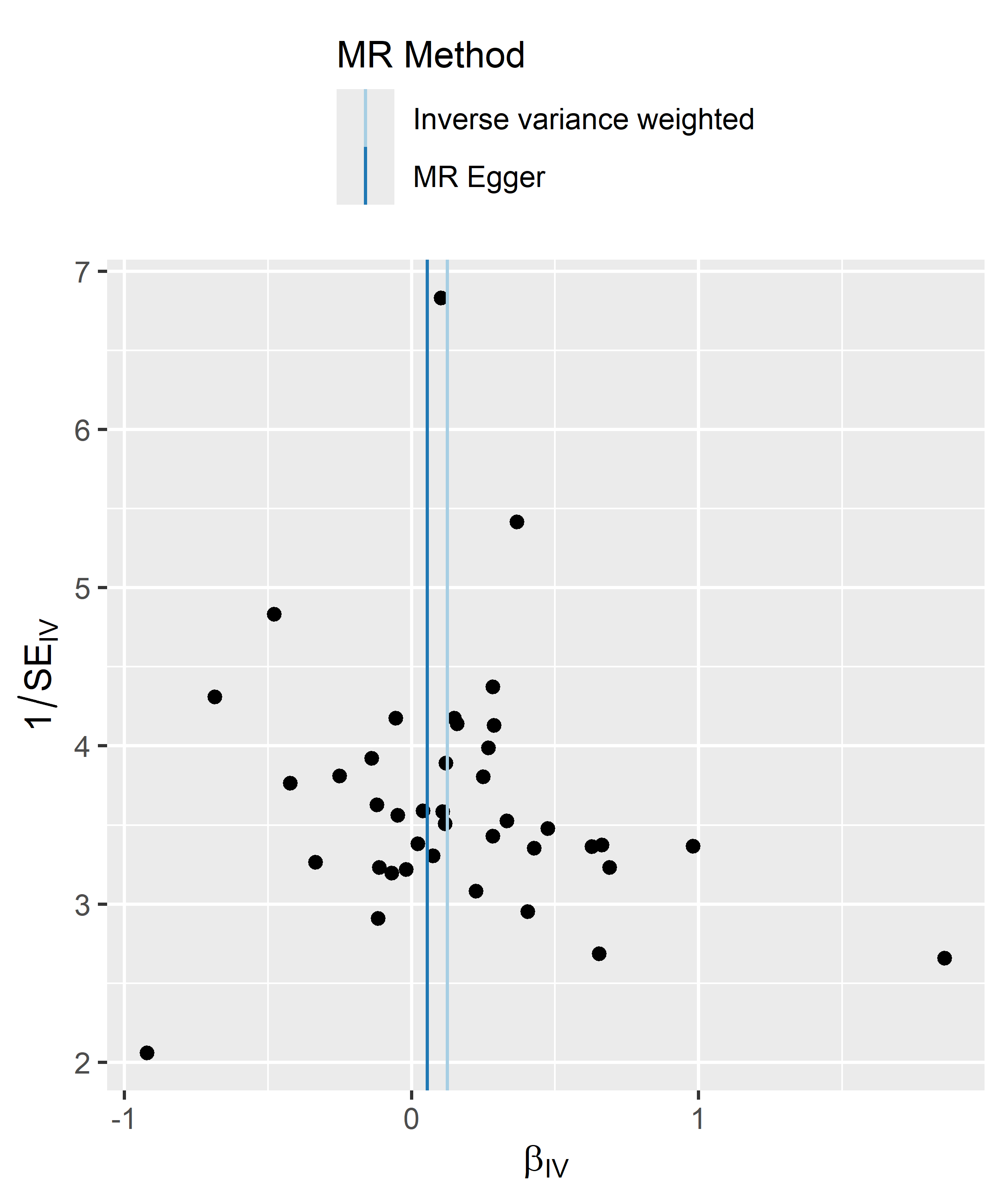


# Tables

## Table S1. Detailed information of GWAS data analyzed in this study.

| **Trait** | **PMID** | **YEAR** | **Cases: Controls** | **Sample size** | **SNPs** | **Population** | **Category** |
| --- | --- | --- | --- | --- | --- | --- | --- |
| RARA^a^ | 35043453 | 2022 | / | 88,411 | 8,489,912 | European | continuous |
| IHD^b^ | / | 2024 | 1:5 | 500,348 | 21,327,062 | European | Binary |

^a^RARA: rest-activity rhythm amplitude.

^b^IHD: ischemic heart disease.

## Table S2. The information source of covariates.

| Variable | Touchscreen questionnaire | | Hospital records | Death Registry | Accelerometer |
| --- | --- | --- | --- | --- | --- |
|  | Initial assessment | Repeated assessments |  |  |  |
| Age at accelerometry | Field ID 34&52 | - | - | - | Field ID 90010 |
| Sex | Field ID 31 |  |  |  |  |
| Ethnicity | Field ID 21000 |  |  |  |  |
| TDI | Field ID 189 |  |  |  |  |
| Recruitment regions | Field ID 54 |  |  |  |  |
| Educational attainment | Field ID 6138 |  |  |  |  |
| Alcohol Status | Field ID 1558 | Field ID 1558 |  |  |  |
| Smoke Status | Field ID 20116 | Field ID 20116 |  |  |  |
| Healthy diet^1^ | Field ID 1309, 1319,1289, 1299,1329, 1339,1349, 1369,1379, 1389,1438, 1448,1458, 1468 | Field ID 1299,1329, 1339,1349, 1369,1379, 1389,1438, 1448,1458, 1468 |  |  |  |
| BMI | Field ID 21001 | Field ID 21001 |  |  |  |
| IHD_PRS | Field ID 26227 |  |  |  |  |
| Season at start time |  |  |  |  | Field ID 90010 |
| Recruitment region | Field ID 54 |  |  |  |  |
| Diabetes history | Field ID 2443 | Field ID 2443 | ICD-10: E10:E14 | ICD-10: E10:E14 |  |
| Lost to follow-up | Field ID 190&191 |  |  |  |  |
| IHD | Field ID 20002 | Field ID 20002 | ICD-10: I20:I25 | ICD-10: I20:I25 |  |
| Hypertension history | Field ID 20002 | Field ID 20002 | ICD-10: I10:I15 | ICD-10: I10:I15 |  |
| RARA |  |  |  |  | Field ID 90004 |

^Healthy diet: According to the Dietary Guidelines for Americans (DGA) recommendation, a healthy eating pattern that includes specific amount of consumption for a variety of fruits, vegetables, fish, lean proteins (red and processed meat), whole and refined grains, and low-fat dairy products.^ ^Healthy diet category was defined to include at least 4 of the following 7 food groups:^ ^1. Fruits: ≥ 3 servings/day, 2. Vegetables: ≥ 3 servings/day, 3. Fish: ≥ 2 servings/week, 4. Processed meats: ≤ 1 serving/week, 5. Unprocessed red meats: ≤ 1.5 servings/week, 6. Whole grains: ≥ 3 servings/day. 7. Refined grains: ≤1.5 servings/day^

## Table S3. Sensitivity analyses for associations of RARA with risks of incident IHD.

| Sensitivity strategy^1^ | HR (95% CI) | *P* | E value |
| --- | --- | --- | --- |
| Manuscript | 1.20 (1.12-1.30) | <.001 | 1.69 |
| Strategy 1 | 1.19 (1.10-1.29) | <.001 | 1.67 |
| Strategy 2 | 1.21 (1.12-1.30) | <.001 | 1.71 |
| Strategy 3 | 1.26 (1.11-1.44) | <.001 | 1.83 |
| Strategy 4 | 1.13 (1.03-1.24) | .01 | 1.51 |
|  | 1.31 (1.19-1.45) | <.001 | 1.95 |
| Strategy 5 |  |  |  |
| Chronic IHD | 1.29 (1.19-1.39) | <.001 | 1.90 |
| Acute IHD | 1.12 (1.02-1.24) | .02 | 1.49 |
| Strategy 6 | 1.21 (1.12-1.30) | <.001 | 1.71 |
| Strategy 7 | 1.20 (1.12-1.29) | <.001 | 1.69 |
| Strategy 8 | 1.24 (1.13-1.36) | <.001 | 1.79 |

Sensitivity strategy^1^: Strategy 1: individuals diagnosed with IHD within 1 year were excluded to minimize the potential of reverse causality. Strategy 2: the analyses were restricted to European whites. Strategy 3: analyses were repeated by defining low-RA as a cutoff of more than 2 standard deviations below the mean. Strategy 4: analyses were repeated by using tertiles to transform RA into a 3-category variable. We computed the E-value to assess potential unmeasured confounders for all models. Strategy 5: a subgroup sensitivity analysis to explore the association between RARA and IHD varies between acute and chronic conditions. Strategy 6: a sensitivity analysis using only hospital records and death registry data to define outcomes. Strategy 7: a sensitivity analysis further adjusting for baseline characteristics such as chronic kidney disease and family history of cardiovascular disease. Strategy 8: we censored data up to Dec 31, 2019 (to account for the start of the COVID-19 pandemic)

## Table S4. Characteristics of genetic variants used to estimate the effect of RARA on IHD.

| Snp | Chr | Pos | | Beta. exposure | | Se. exposure | Beta. outcome | Se. outcome | Effect  allele.  exposure | Other  allele.  exposure | Effect  allele.  outcome | Other  allele.  outcome | *P* | *F* |
| --- | --- | --- | --- | --- | --- | --- | --- | --- | --- | --- | --- | --- | --- | --- |
| rs10044063 | 5 | | 64018598 | 0.0226688 | 0.00496846 | | 0.00509602 | 0.00735324 | T | C | T | C | 0.48829 | 20.82 |
| rs10875606 | 5 | | 144390051 | 0.024492 | 0.00491671 | | 0.00811058 | 0.00694256 | C | A | C | A | 0.24271 | 24.81 |
| rs10930874 | 2 | | 179440469 | 0.0302194 | 0.00676147 | | -0.00167537 | 0.00723511 | T | C | T | C | 0.816878 | 19.98 |
| rs11020171 | 11 | | 93123360 | 0.022273 | 0.0049552 | | 0.00164794 | 0.00673558 | C | A | C | A | 0.806718 | 20.20 |
| rs112767081 | 6 | | 4281420 | -0.0523814 | 0.0115189 | | -0.0248339 | 0.0150562 | C | A | C | A | 0.0990627 | 20.68 |
| rs113851554 | 2 | | 66523432 | -0.0823589 | 0.0105768 | | -0.00848978 | 0.0120513 | G | T | G | T | 0.481139 | 60.63 |
| rs113975274 | 22 | | 38836918 | 0.0835231 | 0.0188161 | | -0.0769202 | 0.040534 | G | A | G | A | 0.0577391 | 19.70 |
| rs12328736 | 2 | | 13581852 | 0.026998 | 0.00566366 | | -0.00677895 | 0.00708449 | A | G | A | G | 0.338632 | 22.72 |
| rs12738703 | 1 | | 210416166 | 0.0207749 | 0.00464292 | | 0.013055 | 0.0061748 | C | T | C | T | 0.0344953 | 20.02 |
| rs12956748 | 18 | | 39835760 | 0.0229924 | 0.00501979 | | -0.000440382 | 0.00714122 | T | C | T | C | 0.950828 | 20.98 |
| rs1345431 | 16 | | 49592944 | 0.0206654 | 0.0046735 | | -0.00142473 | 0.00646508 | T | G | T | G | 0.825581 | 19.55 |
| rs1355619 | 9 | | 95507889 | -0.0250892 | 0.00499987 | | 0.00348474 | 0.00639636 | A | G | A | G | 0.585891 | 25.18 |
| rs139906620 | 4 | | 74799098 | 0.0876305 | 0.0179805 | | 0.0572236 | 0.0326233 | C | T | C | T | 0.0794182 | 23.75 |
| rs142919395 | 8 | | 131408034 | 0.0976401 | 0.0209925 | | 0.0155279 | 0.0235834 | T | C | T | C | 0.510265 | 21.63 |
| rs148611881 | 22 | | 31516436 | 0.0708591 | 0.0154532 | | 0.0259868 | 0.0130831 | C | T | C | T | 0.0470024 | 21.03 |
| rs153685 | 5 | | 88685213 | 0.0247748 | 0.00481443 | | 0.0029442 | 0.0063635 | C | T | C | T | 0.6436 | 26.48 |
| rs1668855 | 18 | | 24949837 | 0.021087 | 0.00453829 | | 0.000453372 | 0.00623507 | T | C | T | C | 0.942034 | 21.59 |
| rs17727577 | 8 | | 51046552 | -0.0418887 | 0.00886004 | | -0.0169393 | 0.014185 | C | T | C | T | 0.232411 | 22.35 |
| rs1940599 | 18 | | 5888816 | -0.0263642 | 0.00518054 | | -0.00706829 | 0.00661057 | T | C | T | C | 0.284961 | 25.90 |
| rs2136614 | 10 | | 62941526 | -0.0330598 | 0.00646217 | | -0.0035781 | 0.00922169 | A | G | A | G | 0.698009 | 26.17 |
| rs28972181 | 13 | | 113970643 | -0.0220603 | 0.00460494 | | -0.0025796 | 0.00628592 | A | C | A | C | 0.681528 | 22.95 |
| rs55904513 | 3 | | 23829192 | -0.0677381 | 0.0152884 | | -0.00273385 | 0.0188731 | G | A | G | A | 0.884826 | 19.63 |
| rs61801633 | 1 | | 168837015 | -0.027801 | 0.0057355 | | 0.00930067 | 0.00851334 | G | A | G | A | 0.274621 | 23.50 |
| rs62296310 | 4 | | 11738558 | -0.0308642 | 0.00680869 | | -0.00884286 | 0.00747195 | A | G | A | G | 0.236621 | 20.55 |
| rs6509018 | 19 | | 42540177 | -0.0212772 | 0.00480758 | | -0.0208391 | 0.00632065 | T | G | T | G | 0.000977305 | 19.59 |
| rs6938671 | 6 | | 530476 | -0.0253132 | 0.00518189 | | 0.0107123 | 0.00672156 | G | T | G | T | 0.110999 | 23.86 |
| rs7219298 | 17 | | 61247054 | -0.0488809 | 0.0101658 | | 0.0059176 | 0.0134689 | T | G | T | G | 0.660406 | 23.12 |
| rs72804375 | 5 | | 152291866 | -0.0303184 | 0.00602583 | | -0.00756946 | 0.00796407 | T | C | T | C | 0.341883 | 25.32 |
| rs72936986 | 2 | | 158572867 | 0.0226859 | 0.00509056 | | 0.00967186 | 0.00676173 | A | C | A | C | 0.152607 | 19.86 |
| rs75818601 | 1 | | 175019430 | -0.0302741 | 0.00535615 | | -0.00854236 | 0.00691948 | C | T | C | T | 0.217003 | 31.95 |
| rs7675318 | 4 | | 19737209 | 0.0344363 | 0.00600601 | | 0.00511269 | 0.00824488 | C | T | C | T | 0.535189 | 32.87 |
| rs78030362 | 19 | | 18464383 | -0.0446962 | 0.00871488 | | -0.0829843 | 0.0168123 | A | G | A | G | 7.98E-07 | 26.30 |
| rs8060241 | 16 | | 61730067 | 0.029495 | 0.0066354 | | 0.0203335 | 0.00912573 | T | C | T | C | 0.0258702 | 19.76 |
| rs926914 | 22 | | 41022150 | -0.0234524 | 0.0050466 | | 0.00113425 | 0.00658284 | C | T | C | T | 0.863199 | 21.60 |
| rs9377155 | 6 | | 148731894 | -0.0294798 | 0.00638748 | | 0.00346134 | 0.0101279 | T | C | T | C | 0.732529 | 21.30 |
| rs9648583 | 7 | | 5871660 | -0.0251936 | 0.00550395 | | 0.00282975 | 0.00779265 | G | A | G | A | 0.716508 | 20.95 |
| rs9817730 | 3 | | 131888811 | -0.0233666 | 0.0050855 | | -0.0154928 | 0.00692628 | G | A | G | A | 0.0252982 | 21.11 |
| rs9818758 | 3 | | 49345492 | -0.0363281 | 0.00606352 | | 0.017395 | 0.00751477 | G | A | G | A | 0.0206253 | 35.90 |
| rs9844835 | 3 | | 140260258 | -0.0222847 | 0.00469411 | | -0.00630227 | 0.00649508 | C | T | C | T | 0.331889 | 22.54 |
| rs9922959 | 16 | | 86799077 | -0.0536864 | 0.0120561 | | 0.0368123 | 0.0124519 | G | T | G | T | 0.00311293 | 19.83 |

## Table S5. The additive interaction of RARA and IHD-PRS with IHD in the UK Biobank Study.

| Compare level | Indices | Estimates | 95% CI | P |
| --- | --- | --- | --- | --- |
| For intermediate IHD-PRS:  HR_00_, HR_01_, HR_10_, HR_11_ | RERI^a^ | -0.01 | -0.27 to 0.25 | 0.53 |
|  | AP^b^ | -0.01 | -0.15 to 0.14 | 0.47 |
|  | SI^c^ | 0.99 | 0.71 to 1.38 | 0.53 |
| For high IHD-PRS:  HR_00_, HR_02_, HR_10_, HR_12_ | RERI | 0.10 | -0.26 to 0.47 | 0.29 |
|  | AP | 0.04 | -0.10 to 0.18 | 0.28 |
|  | SI | 1.07 | 0.85 to 1.35 | 0.29 |

^a^RERI, the relative excess risk due to interaction; ^b^AP, the proportion of disease among those with both exposures that is attributable to their interaction; ^c^SI, the synergy index. HR_00_: high RARA and low IHD-PRS, HR_01_: high RARA and intermediate IHD-PRS, HR_10_: low RARA and low IHD-PRS, HR_11_: low RARA and intermediate IHD-PRS, HR_02_: high RARA and high IHD-PRS, HR_12_: low RARA and high IHD-PRS.
